# Identification of Building Damage from UAV-Based Photogrammetric Point Clouds Using Supervoxel Segmentation and Latent Dirichlet Allocation Model

**DOI:** 10.3390/s20226499

**Published:** 2020-11-13

**Authors:** Chaoxian Liu, Haigang Sui, Lihong Huang

**Affiliations:** State Key Laboratory of Information Engineering in Surveying, Mapping and Remote Sensing, Wuhan University, Wuhan 430079, China; cx_leo@whu.edu.cn (C.L.); lhhuang1018@whu.edu.cn (L.H.)

**Keywords:** earthquake, building damage, photogrammetric point cloud, supervoxel segmentation, latent Dirichlet allocation model, random forest

## Abstract

Accurate assessment of building damage is very important for disaster response and rescue. Traditional damage detection techniques using 2D features at a single observing angle cannot objectively and accurately reflect the structural damage conditions. With the development of unmanned aerial vehicle photogrammetric techniques and 3D point processing, automatic and accurate damage detection for building roof and facade has become a research hotspot in recent work. In this paper, we propose a building damage detection framework based on the boundary refined supervoxel segmentation and random forest–latent Dirichlet allocation classification. First, the traditional supervoxel segmentation method is improved to segment the point clouds into good boundary refined supervoxels. Then, non-building points such as ground and vegetation are removed from the generated supervoxels. Next, latent Dirichlet allocation (LDA) model is used to construct the high-level feature representation for each building supervoxel based on the selected 2D image and 3D point features. Finally, LDA model and random forest algorithm are employed to identify the damaged building regions. This method is applied to oblique photogrammetric point clouds collected from the Beichuan Country Earthquake Site. The research achieves the 3D damage assessment for building facade and roof. The result demonstrates that the proposed framework is capable of achieving around 94% accuracy for building point extraction and around 90% accuracy for damage identification. Moreover, both of the precision and recall for building damage detection reached around 89%. Concluded from comparison analysis, the proposed method improved the damage detection accuracy and the highest improvement ratio is over 8%.

## 1. Introduction

It is crucial to conduct the accurate assessment of structural damage to buildings after disaster events. Final assessment results are beneficial to immediate relief efforts and subsequent post-disaster reconstruction [1,2]. Traditional ground-based investigations are time-consuming and dangerous. They also require a large amount of labor and material resources. That is because with the development of remote sensing (RS) technology, building damage information can be obtained efficiently. RS offers an efficient way of obtaining building damage information [3,4].

**Figure 1:**
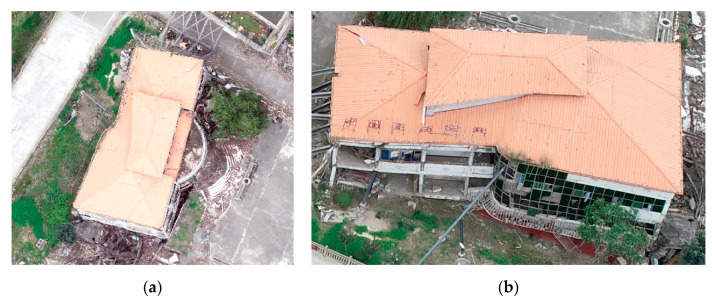
UAV image with (**a**) vertical and (**b**) oblique viewing.

Recently, very high resolution (VHR) optical images, synthetic aperture radar (SAR) data, and light detection and ranging (LiDAR) data provide more detailed damage characteristics in the detection of damaged buildings [5,6,7]. In practical applications, optical data are preferred because they are relatively easy to interpret. With the continuous improvement of RS image resolution, disaster damage assessment can be conducted through various platforms, such as satellites, manned aircraft, and unmanned aerial vehicles (UAVs) [8,9,10]. In addition, with the development of RS platforms, recent research focused on the application of airborne techniques in emergency response [9,11]. Spaceborne RS technology is typically used to discover building damage within a large area. It is an effective method that assures satellite image availability. In contrast, data acquisition methods based on airborne UAVs benefit from flexibility, low cost, and real-time monitoring [12]. Unlike traditional RS observation methods, which only acquire single view image features and plane geometry information of ground objects, UAVs can observe object features from multiple angles. With the development of oblique photography techniques, building facade information can be obtained directly from the UAV platform [13,14]. ‘Intact buildings’ are identified from orthophotos (for example, Figure 1a) is actually inclined and partially collapsed as appear when it is portrayed by UAV images (for example, Figure 1b). Thus, the detection accuracy of structural building damage cannot be assured when detection is merely based on a single view. As such, the UAV oblique observation technique has the potential to improve structural damage detection accuracy with a more detailed facade and roof information.

Apart from new observation techniques, new damage detection methods are being rapidly developed with the aid of artificial intelligence and computer vision [15,16]. Based on the results of latest studies, it can be said that deep-learning algorithms are becoming popular for the detection of building damage and have greatly improved in terms of their detection accuracy [15,16,17]. Deep learning is extremely efficient as in this technology, nonlinear spatial filters can be automatically learned and a hierarchy of increasingly complex features can be directly generated from the original data. Furthermore, deep learning has demonstrated superior flexibility and capability compared with the traditional classification methods [18]. Owing to the convolutional neural network (CNN) structure, the entire CNN system alleviates the requirement to design a suitable feature extractor manually. However, deep learning requires several training samples and a long training time owing to its deep CNN structures. Typical damage characteristics may also vary depending on the area and the spatial image resolution, limiting the generalization and application of a specific trained model [19]. Furthermore, considering the complexity of building damages, 3D damage features (i.e., geometric and elevation features) are more useful than 2D image features for damage detection [16,20]. Thus, a multi-feature building damage detection method combining 2D and 3D features is critically needed. Nowadays, several studies use 3D features to classify or detect typical objects, such as buildings, vegetation, cars, and traffic signs [21,22]. Although 3D structural analysis for building can significantly improve the detection accuracy, it has not yet been fully exploited due to the following reasons: (1) the object features are complex and vary for most of the damaged buildings, making it difficult to select typical 3D features of the damaged buildings; (2) structural building damage details (e.g., cracks, local scaling) are difficult to detect with traditional observation methods.

To address these issues, we proposed herein a building damage detection method based on oblique photogrammetric point clouds using supervoxel segmentation and a latent Dirichelet allocation model. Upon selecting the data source, oblique photogrammetric points were used to extract the 3D damage features rather than the LIDAR points. This method has proved to be low cost, with point precision similar to LIDAR technique, and rich in RGB information. For the correct selection and representation of typical features of damaged buildings, we accounted for the complexity and particularity of building damage. Thus, we combined the 2D and 3D features to achieve accurate building damage detection based on supervoxels and an LDA model. We provided a fully automatic and general framework for detecting building damage by effectively overcoming the influence of other ground objects.

The innovative contributions of our proposed approach are as follows: (1) Different from the traditional point-based classification method with low accuracy, we developed one supervoxel-based damage detection method. Considering the generated supervoxels using the classic Voxel Cloud Connectivity Segmentation (VCCS) algorithm suffer from “zig-zag” effect, we developed a refined boundary supervoxelization algorithm. The proposed method consists of detecting and refining the boundary points, which significantly enhances the precision of damage locations. (2) Considering the detection accuracy of structural building damage cannot be assured when detection is merely based on a single view, we combined 2D and 3D features together using the LDA model in this study. The LDA model generalizes these point-based features and builds the representation of high-level features. This new approach provides a systematic view on the efficient and autonomous processing of rooftop and facade features into useful structural damage information. (3) In view of the difficulty in replicating the approach, we provided a general and accurate realization framework combining building point extraction with building damage detection. Such a methodology improves damage-detection accuracy and can be replicated in fine building damage assessment.

## 2. Study Area and Data Sources

**Figure 2:**
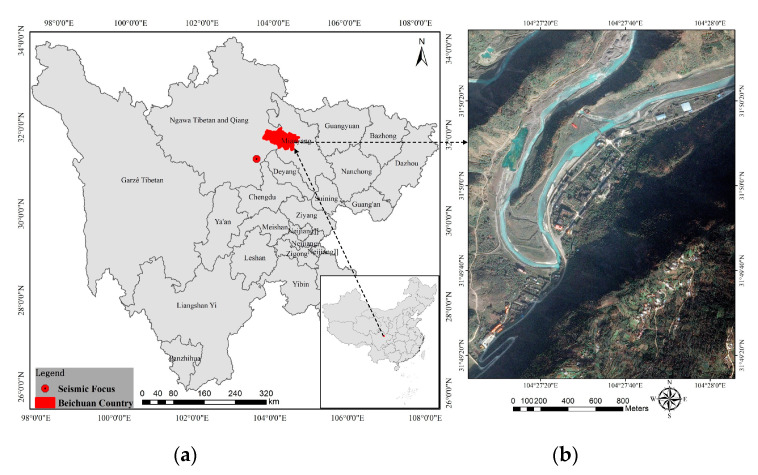
Location map (**a**) and Google map (**b**) of study area.

A violent Ms 8.0 Wenchuan earthquake occurred on 12 May 2008. It killed nearly 70,000 people, injured more than 370,000, left more than 17,000 listed as missing, and destroyed most of the buildings. We selected the old town of Beichuan in Sichuan province, China, as our study area because it was completely preserved as the site of the Wenchuan earthquake. Although the town was largely destroyed due to the strong earthquake, different types of building damage can still be found in this site even after 12 years. Even though the initial damage features are no longer significant, the building damage research at the site is of high value due to the abundant and variable building damage types and damage samples. An overview of the study area, including its spatial location on Google maps, is presented in Figure 2. 

**Figure 3:**
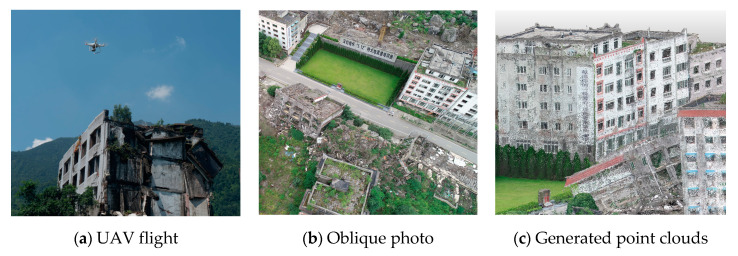
UAV data acquisition and some data products.

To evaluate the effectiveness of our proposed method, we mapped the Beichuan earthquake ruins on the ground during 12–16 August 2019. As a UAV platform, we used DJI-Phantom 4 Pro, which was equipped with a digital camera (8.8–24 mm f/2.8–11 lens, 5456 × 3632 pixel image size) and APS CMOS sensor (25.4 × 25.4 mm). The camera rotated freely, thereby allowing multiple views and taking images with a count of 20 million pixels. The DJI software package, Ground Station, was used for photogrammetric flight mission planning. The flights were performed with a planned side overlap of 80%, and oblique photos were obtained with a dip angle of 55°. To acquire additional damage details with a flying height limitation, we set the flying height to approximately 100 m to achieve a spatial resolution of 1 cm. Considering the large flight area of nearly 15 km^2^ and the battery limitations, the entire study area was divided into five parts. We took overlapped photos from the adjacent regions. More than 1400 digital images (Figure 3b) were collected. These images were imported into the Pix4d software to generate dense point clouds (Figure 3c). To avoid the occurrence of ‘empty holes’ in the final point clouds, formed due to the platform’s instability during flight, we organized multi-group images for each subset area. 

## 3. Methodology

**Figure 4:**
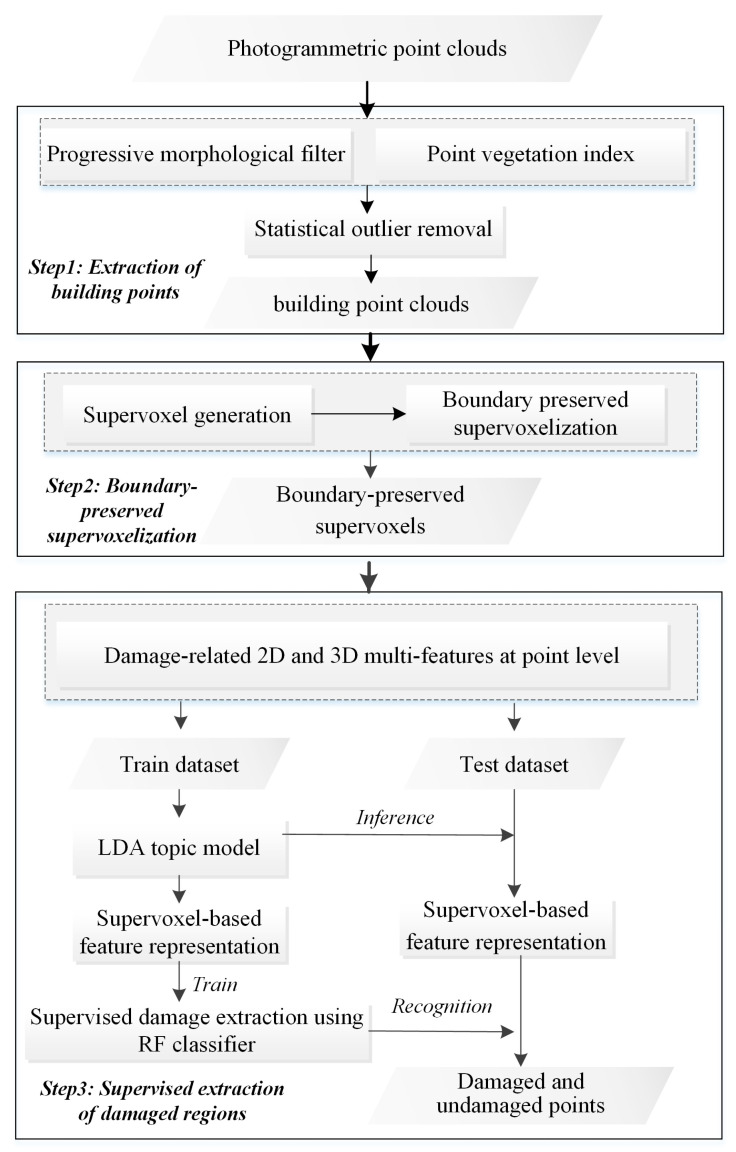
Framework of building damage detection using photogrammetric point clouds.

Our methodology included the following steps: extraction of building points, supervoxel construction, and supervised extraction of damaged regions (Figure 4).

### 3.1. Extraction of Building Points

The premise of identifying building damage after a disaster is to distinguish building regions from various land-cover types. We extracted the building points after masking out the ground vegetation and conducting the statistical outliner removal (Step 1, Figure 4). In this study, ground vegetation and building points were classified at supervoxel level from the post-event UAV-based photogrammetric point clouds by combining point-level extraction and supervoxel-based optimization. The extraction of a specific class was first conducted at the point level. After three land-cover types were extracted, supervoxel-based majority rules were used to produce classification results at the supervoxel level. The supervoxel was identified as a specific class if most points within the object (more than 60%) belonged to that specific class.

#### 3.1.1. Progressive Morphological Filter

The progressive morphological filter (PMF) algorithm was used to remove the non-ground points from the point cloud [23]. Using an open operator with increasing window size, the filter can remove non-ground points grid by grid. In this method, the elevation difference threshold *D_T,k_* is calculated according to the change of adjacent window size and terrain slope, as shown in Equation (1).

(1) $$D_{T,k}\;=\;\{\begin{array}{c} \;\;\;\;\;\;D_{h},\;\;\;\;\;\;if\;w_{k}\leq 3\;\;\;\;\;\;\;\;\\ s\left(w_{k}-w_{k-1}\right)c+D_{h},\;\;\;if\;w_{k}\leq 3\;\;\;\\ \;\;D_{hmax},\;\;\;if\;D_{T,k}>D_{hmax} \end{array}$$

where *w_k_* is the window size of *k*th filter; *D_h_* is the initial elevation difference threshold; *s* is the slope; *c* is the cell size; and *D_hmax_* is the maximum elevation difference threshold. Thus, different windows correspond to different thresholds. By comparing the elevation difference before and after the point cloud operation, the points whose height difference change is greater than the threshold value are determined as non-ground points and filtered out. Iterations are continued until the size of the filtering window was greater than the previously defined maximum threshold. In conclusion, the adopted approach takes full into account different terrain conditions using slope factor, and can be used in both the urban and mountain areas [24]. 

By setting *D_h_* very low, PMF contributed to the detection of regions with debris, which could be misclassified as ground points due to a similar elevation. After several tests, the initial elevation difference threshold directly affected the extraction of debris. Points with a height above the estimated ground surface of more than *D_h_* were classified as non-ground points. Based on the statistical results of the point cloud error (0.22 m) and vertical accuracy (0.14 m), optimal threshold *D_h_* was determined by referring to the lower value (0.14 m). In this study, the PMF algorithm was run together with the Point Cloud Library.

#### 3.1.2. Point Vegetation Index

Vegetation was extracted based on the RGB features using the calculated point vegetation index (PVI), as shown in equation (2), where R, G, and B represent the red, green, and blue channel, respectively. The specific constants were determined following [25]. Unlike the traditional normalization difference vegetation index (NDVI), which requires near-infrared information, PVI only requires the RGB information. A point with a PVI value higher than selected threshold *P_h_* (0.03) was identified as vegetation; otherwise, it was identified as non-vegetation. The optimal segmentation threshold was determined using the Ostu method. Ostu was based on the adaptive threshold selection and maximization of interclass variance. By statistical analysis of histogram features of the whole image, the optimal global threshold was determined as

PVI = (2G − R − B)/(2G + R + B)(2)

#### 3.1.3. Statistical Outlier Removal

After masking out the ground and vegetation points, the isolated points were likely outliers. The statistical outlier removal (SOR) algorithm was used to remove outlier points that were further away from their neighbors than the average for that point cloud, including vehicles, people, and undetected vegetation points [26,27]. After SOR processing, the remaining points were regarded as the final building point clouds would be used for the follow-up study. Based on the mean Euclidean distance between each point and its closest neighbors, statistics of the mean distances were applied to characterize the distribution across all points in the cloud. Specifically, we calculated the mean and standard deviations. We removed the extracted statistical outliers based on those univariate values using Equation (3)

(3) $$P*\;=\;\{\rho \epsilon P|d\leq \left(\mu_{k}+\gamma \cdot \sigma_{k}\right)\},$$

where P* is the entire point cloud after the statistical outlier removal; *μ_k_* and *σ_k_* represent the mean and standard deviation of the Euclidean distance between each point and its *k-*closest neighbors, respectively; and γ is the scalar multiplier that controls the neighboring distance for the point removal. After repetitive testing experiments, γ is set to 0.4 in this study.

### 3.2. Boundary Refined Supervoxel Segmentation 

Similar to superpixel segmentation in 2D image processing, supervoxel segmentation tends to be used to divide the point cloud into simple yet meaningful segments. It provides a salient and distinctive local geometric representation of 3D points and enables the operations to be performed on regions rather than on scattered points [28]. Generally, supervoxel segmentation is achieved using the VCCS algorithm [29]. However, the generated supervoxels of VCCS cause the “zig-zag” pattern with uneven edges [30]. To overcome this problem, we adopted a boundary refined supervoxel supervoxelization method, which consisted of supervoxel generation and boundary refinement (Step 2, Figure 4).

#### 3.2.1. Supervoxel Generation

To reduce the number of points, 3D points were first grouped to generate a voxel. Then, voxels were clustered to form supervoxels based on the spectral and geometrical relationships of voxels in the 3D space. The VCCS algorithm was used to produce a 3D point cloud supervoxel based on modified k-means clustering and a local iterative clustering algorithm, which used geometric and color attributes. In particular, seed voxels were first clustered into supervoxels based on the selected seed resolution. Then, supervoxel attributes were initialized for every seed voxel comprising the color and geometric properties of the voxel. From the limited search space, we calculated the distance from the center of supervoxels to the neighboring voxels using Equation (4)

(4) $$D_{1}=\sqrt{\frac{\lambda D_{c}^{2}}{m^{2}}+\frac{\mu D_{s}^{2}}{3R_{s}^{2}}+\varepsilon D_{f}^{2},}$$

where, *D*_1_ is the distance between the center of supervoxels and the neighboring voxels; *D_c_* is the normalized Euclidean color distance; *D_s_* is the normalized spatial distance for clustering; *R_s_* is the seed resolution; and *D_f_* is the distance on the fast point feature histogram (FPFH) space computed using the histogram intersection kernel. In addition, *λ*, *µ*, and *ε* represent the influence degree that determine the color, spatial, and geometric relationships between the voxels, respectively. The color and spatial distances would be assigned equivalent weighting, while the weight of the FPFH distance was slightly higher. Voxels would be assigned to adjacent supervoxel centers by distance measurement to update the centroid of the supervoxels. This process continued until the searching of adjacent points for supervoxel construction stopped, and the supervoxels centroids were stabilized. 

#### 3.2.2. Boundary Refined Supervoxelization 

Our proposed method for boundary refined supervoxel generation consist the detection of boundary points, and the refinement of boundary points. Firstly, based on the generated VCCS supervoxel, points within each supervoxel were defined by the distance from the point to the supervoxel center considering the local curvature exploring the spatial proximity of adjacent supervoxels in geodetic space [31]. If the distance was larger than a given threshold, which was empirically set to be one-half of the seed supervoxel resolution, the point was regarded as a boundary point. The radius size of the spherical neighborhoods for estimating the normal vector was equal to the supervoxel size. Then, we conducted local k-means clustering based on the boundary point and the centers of neighboring supervoxels. As Equation (5) showed, the whole clustering process was governed by a distance measurement considering the normal vectors and spatial distance.

(5) $$D_{2}=\sqrt{w_{n}\cdot {\left|\left|N_{i}-N_{b}\right|\right|}_{2}+w_{d}\cdot {\left|\left|P_{i}-P_{b}\right|\right|}_{2},}$$

where, *D*_2_ is the distance between the boundary point and the neighboring supervoxel centers; *N_i_* is the normal vector of the center of one neighboring supervoxel; *N_b_* is the normal vector of the boundary point; and *P_i_* and *P_b_* are their positions on the tangent plane defined by the normal vector *N_i_*. In addition, *w_n_* and *w_d_* denote the weight factors for the angle between normal vectors and the distance between centroids, respectively. Empirically, they were set to 1 and the reciprocal value of the voxel size, respectively.

**Figure 5:**
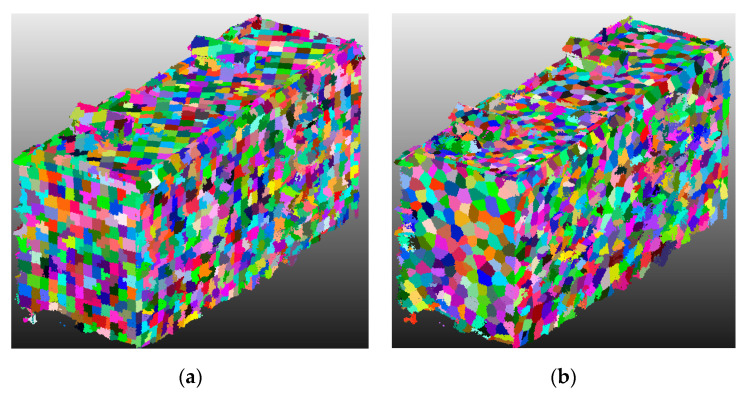
Examples of VCCS supervoxels (**a**) and boundary refined supervoxels (**b**).

As for the key parameters, the voxel size was set to 0.2 m, and the seed resolution of supervoxels was set to 0.8 m. As shown in Figure 5, there are great differences between the supervoxels using the VCCS algorithm and our boundary refined algorithm. A ‘zig-zag’ effect is very obvious for final boundary between supervoxels using the traditional VCCS algorithm. In contrast, the boundary between supervoxels using the proposed method is obviously smoother, which can reflect the real boundary between different objects to a certain extent.

### 3.3. Supervised Extraction of Damaged Building Regions 

We conducted the supervised extraction of building damage based on 2D features, 3D features, and the LDA model (Step 3, Figure 4). First, we extracted different features of each point, including color features, texture features, and geometric features. The features were derived from the 2D images and discrete 3D point clouds. These point-based features were then used to generate more discriminative features of supervoxels at a high level. Finally, a supervised method was used to extract the damaged regions. 

#### 3.3.1. Damage-Related 2D and 3D Multi-Features at Point Level

1. 2D features

Spectral and textural differences usually form the basis of 2D building damage detection [32,33]. The combination of 2D image features and 3D point features is characterized by accurate space registration for the image and point cloud. Based on the strict geometric model for oblique photos and photogrammetric point clouds, the collinear equation is used to register them [34]. Point clouds are commonly used as a reference to correct the intrinsic and extrinsic parameters of those photos. To accelerate the proceeding speed, we first constructed an image mosaic for the UAV images obtained in the same flight route. Then, we addressed the following steps: (1) registration of mosaic images and point clouds; (2) calculation of 2D features from mosaic images; (3) selection of minimum or maximum feature value for each point with the same spatial location from different images. Considering that the same point may be visible from multiple oblique images of different flight routes, we used the maximum value from all of the selected mosaic images at the specific pixel as the optimal value to represent the unique damage feature.

(a) Hue and saturation: In contrast to the original RGB values, the hue-saturation-value (HSV) color space is less sensitive to illumination changes. The HSV color space separates the luminance and brightness components from the hue, which indicates it is more advantageous for extracting color features. In addition, due to the enhancement of diffuse reflection for damaged building regions, they are generally represented by dark gray colors, dissimilar to the colors of intact building regions. 

(b) Gray level co-occurrence matrix (GLCM)-based texture features: The image texture of intact buildings is uniform, whereas the texture structure of damaged buildings appears broken, disordered, and inconsistent. GLCM is a statistical analysis method for describing regional texture and was used herein to extract the texture features. Similar to Wei and Yang [35], we applied only two effective features in this study: angular second moment (ASM) and entropy (ENT). We used ASM to describe the uniformity of gray-scale distribution and ENT to describe the amount of information.

2. 3D features

(a) Eigenvalue-based feature: Eigenvalues represent the 3D ellipsoid form along its principal axes. The axes provide additional features and help discriminate between the planes, edges, corners, lines, and volumes. For each point, its neighbors within a series of radii are searched, and their covariance features are derived from the local covariance matrix. Considering the neighborhood size directly affects the value of these geometric features, Shannon entropy was used to determine the suitable size [36]. The Shannon entropy was maximized across the increasing K-nearest neighbor with an interval of 10 points and ranged from 50 to 300. Then, we established the optimal neighborhood size (120 points) to compute the covariance eigenvalues and construct 3D geometric features. Linearity (L_λ_), planarity (P_λ_), scattering (S_λ_), and omnivariance (O_λ_) were applied according to their effectiveness in damage detection, as determined from the latest research [16,37], where λ_1_, λ_2_, and λ_3_ represent the covariance matrix’s three eigenvalues (λ1, λ2, and λ3, λ1 ≥ λ2 ≥ λ3 > 0).

L_λ_ = (λ_1_ − λ_2_)/λ_1_, (6)

P_λ_ = (λ_2_ − λ_3_)/λ_1_ (7)

S_λ_ = λ_3_/λ_1_ (8)

(9) $$O_{\lambda }\;=\;\sqrt[{3}]{\left(\lambda_{1}*\lambda_{2}*\lambda_{3}\right)}$$

(b) FPFH feature: FPFH attempts to find surface variations by exploring the relationships between point’s k-neighbors and their estimated surface normal. Through representations of the geometrical properties around a specific point in a high-dimensional space, FPFH provides an informative signature for the feature representation. An FPFH descriptor can depict 3D point clouds effectively, as has been previously proven by other researchers [21,22]. The outcome of FPFH at a point is a multi-dimensional histogram, which describes and generalizes the local curvature at this point. For different types of geometric surfaces, FPFH exhibits different distribution characteristics. In this study, the FPFH descriptor was selected as a component feature for damage detection.

(c) Other features: Apart from the above-mentioned common spectral and geometric features, other features can be used in damage detection. The normal vector for intact buildings, including the roof and facade components, is fixed. However, for damaged buildings (i.e., collapsed and inclined buildings), the normal vector varies. Herein, we used the Z component of the normal vector *(N_z_*) to identify the damaged regions. Some damaged roof or facade were represented by ‘empty holes’. Although we could not directly build point features for such regions, we utilized the surrounding points clustered by the VCCS algorithm to represent relative damage. We also found by visual comparison that the number of points in a segment surrounding these ‘empty holes’ was relatively low. Specifically, in contrast to the intact building region, the point distribution within each supervoxel was more discrete, and the number of points within each supervoxel was small. Thus, the number of points in a supervoxel was defined as the area feature *(N_p_*) and reflected the segment area. For heavily collapsed buildings, elevation difference was also a significant feature. Thus, we used the normalized elevation *(H_n_*) to identify the collapsed regions. This feature demonstrates the difference in elevation between the selected point and the lowest point of the segment.

#### 3.3.2. Supervoxel-Based Feature Representation Using the LDA Model

Generally, point-based low-level visual features cannot provide the effective descriptive ability for object detection. With the development of object-based techniques, multiscale feature construction based on supervoxels has become an effective strategy to extract higher-level semantic features [29]. In this study, the features listed in Section 3.2 were used to form the high-dimensional point descriptor *F_p_*. We also opted for a method different from the traditional one, where the overall feature for each supervoxel is obtained. We utilized a one feature generalizing method based on the LDA model to generalize the supervoxel features and build an optimal feature representation. Specifically, we extracted the vectors of the same length from various supervoxels. Each vector generalized *F_p_* of the points and contained the relation of points in each supervoxel. Moreover, the features should have inherited the advantage of *F_p_*. In contrast to the commonly used bag-of-visual-words (BOVW) approach, which only considers supervoxel as a set of disordered ‘visual words’ and ignores the semantic relationship between the points, the LDA model helped improve the discriminate power of the feature descriptors to a certain extent and reduce its sensitivity to the varying point density. 

**Figure 6:**
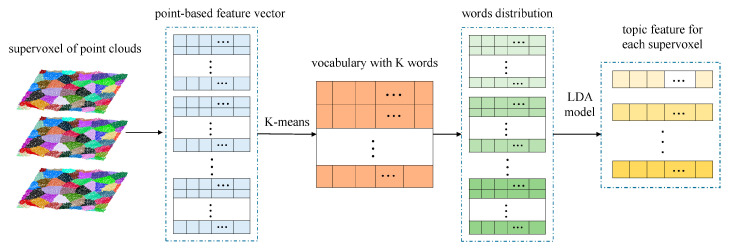
Extraction of supervoxel-based topic feature using LDA model.

Although LDA is commonly used to classify documents and words, the advantage in multiscale feature description allows its application in feature representation of point clouds. In this study, each point-based feature *F_p_* was regarded as a word, and each supervoxel was taken as a document. LDA extracted a certain number of latent topics to represent the main characteristics of a point cluster and describe the documents. Each document was expressed by a vector that consisted of the probability of each latent topic in the corresponding document. As shown in Figure 6, the feature of each supervoxel was expected to be generated as follows:

*Step 1:* Each feature vector of *F_p_* was normalized to the range [−1,1], which could effectively eliminate the numerical difficulties during the feature calculation.

*Step 2*: The fuzzy k-means algorithm was employed to aggregate all point features *F_p_* into k-clusters. After clustering, k-center vectors denoted the word that formed the last dictionary. Then, *F_p_* of each point was represented by a word (cluster center vector) if the distance between the center and the word was the shortest. In addition, the cluster centers were considered the latent topics of the supervoxels.

*Step 3*: By calculating the number of occurrences of the same latent topic within each supervoxel, the frequency vector of the supervoxel could be obtained. In this way, each supervoxel was represented by a frequency vector of the latent topic in the k-dimensional space.

*Step 4*: Based on the frequency vectors, the LDA model was used to obtain the features of each supervoxel, which consists of the probability of each latent topic.

#### 3.3.3. Damage Extraction Based on RF Classifier

Upon completing the data processing, as described in Section 3.2, each supervoxel was assigned a more discriminative feature representation at a higher level and a reference label. The derived normalized feature vectors served as input to a binary supervoxel-based classification in order to distinguish between the ‘damaged points’ and ‘undamaged points’. Considering the classification accuracy and efficiency, a random forest (RF) algorithm, which was a combination of trees-structured classifiers, was used in this study [38]. For non-expert users, RF algorithm is easy to use and tune. Compared with other classifiers, the RF classifier stands out in noise elimination and fast data processing. In addition, it can also achieve an estimate of internal errors [39]. Using the cross-validation method, the optimal RF parameters can be obtained.

## 4. Experiments

In our experiments, the proposed building damage extraction framework was tested using Microsoft Visual C++ (embedding PCL1.8.1) and PyCharm 2019. All of the experiments were carried out on a PC with 40 GB memory and Intel Xeon E5 Central Processing Unit (CPU) with 2.7 GHz. It is worth noting that a sensitivity analysis has been conducted for those significant parameters. Based on final sensitivity analysis results, the optimal majority percent was set to 75%, the optimal supervoxel resolution was determined to be 1.4 m, the optimal number of latent topics and visual words were set to 40 and 400, respectively. As for the RF algorithm, the optimal number of trees and depth were set to 50 and 30, respectively.

### 4.1. Experimental Dataset

**Figure 7:**
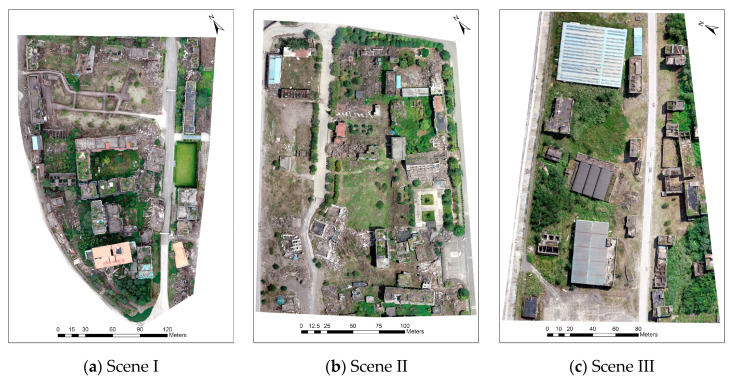
Selected different damaged scenes for test (**a**,**b**) and validation (**c**).

In this experiment, to illustrate the applicability of the proposed method for different types of damage conditions and present more damage details, we applied the proposed method in different damage scenarios: (1) mostly intact buildings with slight damage, (2) mostly collapsed buildings, and (3) buildings with moderate damage. These three scenarios are presented in Scene I in Figure 7a, Scene II in Figure 7b, and Scene III in Figure 7c.

### 4.2. Training Sample Collection

**Figure 8:**
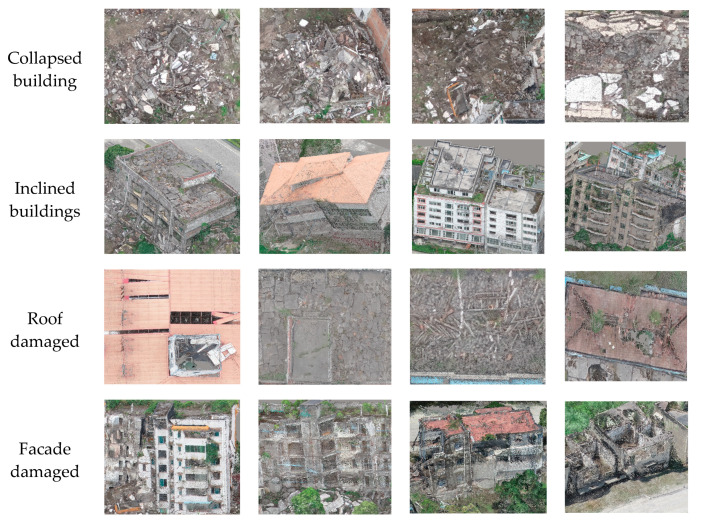
Representative damage samples based on different damage features.

The LDA and RF classifiers were obtained in the training process and applied to classify the unlabeled point clouds. To train the RF classifier, we considered that a sufficient number of representative training examples were required, and that an unbalanced distribution of training examples per class might have a detrimental effect on the training process. Hence, after masking out the ground, high vegetation, and other ground point clouds, we selected as many points as possible for damaged and undamaged building regions outside of the test scenes in the study area with roughly equal numbers, covering as many damage types as possible. Then, we combined these two types of labeled samples from different scenes and trained the classifier. Considering the diversity of building damage types and referring to the European Macroseismic Scale 1998 (EMS98) [40], we categorized the damaged buildings by damage type: collapsed buildings, inclined buildings, roof damaged buildings, and facade damaged buildings. The selected damage samples mainly referred to the following features in Figure 8.

### 4.3. Evaluation Metric

To evaluate our classification, we compared the obtained results with the manually labeled reference data based on field survey data and visual interpretation results using the 3D points. Precision (Pre.), recall (Rec.), overall accuracy (OA), and F_1_-score were used to evaluate the extraction and classification performance for each scene as per Equations (10)–(13). OA provided under- or over-estimates of damage classification in uneven positive and negative samples. Pre. was used to evaluate the false detection ratio, while Rec. was utilized to evaluate the missed detection ratio. F_1_-score denoted the comprehensive evaluation index of Pre. and Rec. Herein, TP denoted the true positive, i.e., the number of positive samples correctly classified as positive. FP stood for the false positive, i.e., the number of negative samples incorrectly classified as positive. FN was the false negative, i.e., the number of positive samples incorrectly classified as negative, and TN denoted the true negative, i.e., the number of negative samples correctly classified as negative.

Pre. = TP/(TP + FP) (10)

Rec. = TP/(TP + FN) (11)

OA = (TP + TN)/(TP + FN + FP + FN) (12)

F_1_-score = (2 × Pre. × Rec.)/(Pre. + Rec.) (13)

## 5. Results and Discussion

### 5.1. Extraction of Building Points and Accuracy of Evaluation

Building points were extracted after filtering and denoising, as illustrated in Section 3.1. We also considered that totally collapsed building objects, such as debris and rubble piles, belonged to the buildings. As such, we set elevation difference threshold *D_h_* to 0.14 to provide a complete damage assessment of collapsed buildings, including their debris. Debris around the base of the damaged buildings had also to be correctly classified in order to facilitate damage assessment in a later portion of the workflow. In addition, last threshold *P_h_* of PVI was determined to be 0.03, based on the statistics results from the histogram by the Ostu method. 

**Figure 9:**
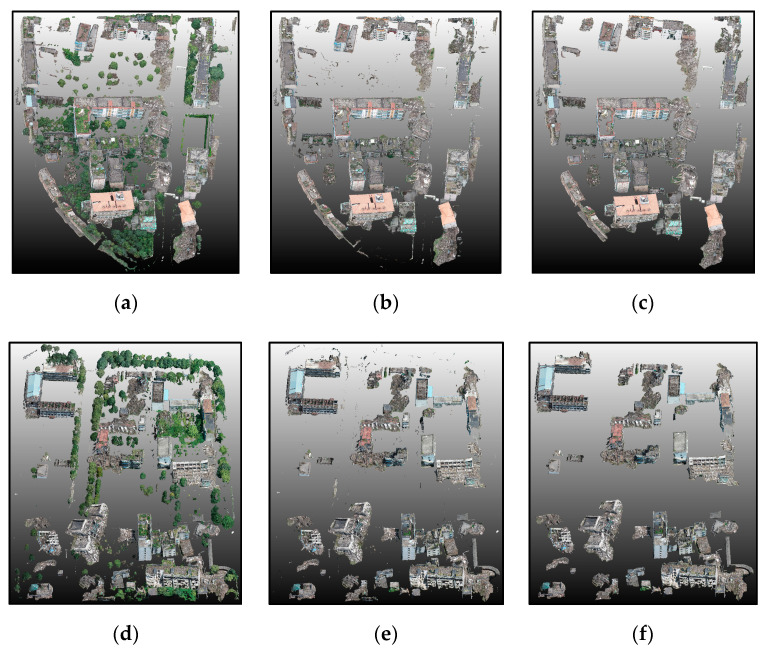
Extraction process and results for building points. (**a**,**d**) represent remaining points after ground filter for Scene I and II. (**b**,**e**) represent remaining points after vegetation removal. (**c**,**f**) represent remaining points after denoising.

After masking out of ground and vegetation points, the remaining points were regarded as building points (Figure 9) and were used in damage extraction. In the accuracy evaluation process, we applied a stratified sampling scheme to select the representative supervoxels and ensure that they were uniformly distributed. For each scene, 3000 damaged and 4000 undamaged supervoxels were compared with the manually labeled reference data based on the field investigation and visual interpretation results. The final accuracy evaluation results are presented in Table 1. Precision, recall, and overall accuracy reached 0.94, demonstrating excellent performance of the proposed method at building point extraction. Based on the step-by-step analysis of the remaining points, two main building point extraction errors resulted from the threshold for *D_h_* and *P_h_*. Due to the long time span after the disaster, few building regions were covered with bryophytes or low vegetation, which caused missed extraction. A small percentage (6%) of missed extraction was due to the presence of low vegetation and bryophyte in the vicinity of the observed buildings. Although there were a few missed and false extractions, the test results met the basic requirements and set a foundation for further study. In terms of time consumption, extracting building points in Scene I and II cost about the similar amount of time, despite there being a much larger number of points in Scene II. The final result demonstrated the validity and efficiency of the proposed method.

### 5.2. Identification of Damaged Building Points and Accuracy of Evaluation

In the previous sections, we extracted 16,528,693 and 18,269,569 building points from Scene I and Scene II, respectively. After the extraction of building points, supervoxel segmentation was conducted for each scene, with a total of 41,925 supervoxels. Based on the supervoxels from Scene I and II, 4126 damaged building supervoxels and 4259 intact building supervoxels (approximately 20%) were randomly selected to learn the LDA model and RF classifier. In addition, for a supervoxel-based RF classifier, we performed a heuristic grid search to define the settings of the RF classifier. We set the number of involved decision trees to 50 for all of the considered feature sets. The LDA-based methods also involved two parameters: *N_topic_* (number of topics) and *N_dictionary_* (number of words). Although the number of trees was set to 50, more trees would have yielded slightly better accuracy but also would have linearly increased the computation cost. The depth of RF was estimated with cross-validation over a parameter from 20 to 40 with a step length of 2. Through trial-and-error experiments, we found that an RF depth of approximately 30 (with the Gini-index as the splitting criterion) yielded the most accurate results. The selection process of these parameters is presented in Parameter Sensitivity Analysis, including the detailed parameter sensitivity analysis. 

**Figure 10:**
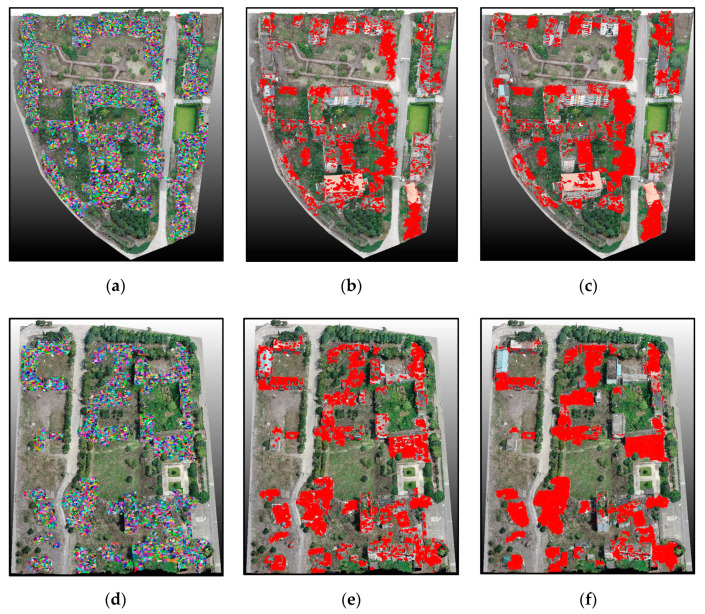
Supervoxel segmentation and damage identification results for test scenes. (**a**,**d**) represent supervoxel segmentation results. (**b**,**e**) represent classified damaged regions. (**c**,**f**) represent reference results.

**Figure 11:**
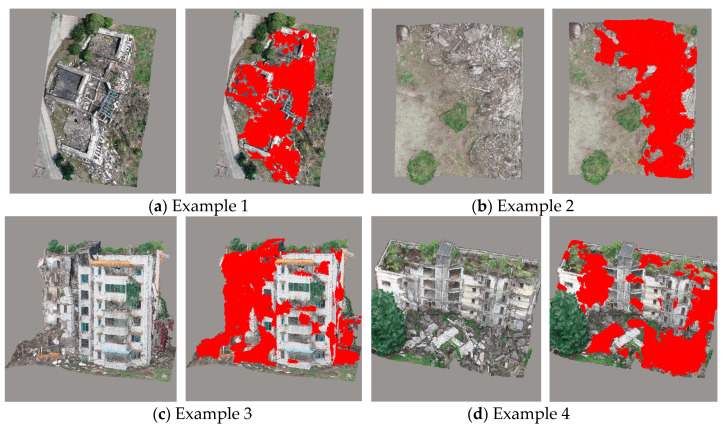

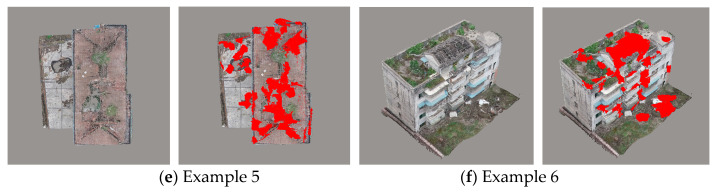
Some examples for building damage extraction.

Based on the learned LDA and RF classifiers, the identification of damaged building regions was conducted for Scene I and Scene II. As can be deduced from the final global classification results (Figure 10) and a local area enlargement (Figure 11), with the red regions indicating damaged rooftop areas, the extracted results substantially covered the true damaged building regions and demarcated roughly the position of damaged regions (e.g., facades, roofs), despite some false and missed extraction results. Moreover, the proposed method showed great applicability for damage detection of different types, including debris, spalling, rubble pile, and ‘hole areas’. 

For quantitative validation, extraction results were compared with the manually labeled reference data based on the visual interpretation results, including 10,000 damaged and undamaged samples. The accuracy indices are listed in Table 2. The results show that the damage detection technique for building areas performed well (average OA: ~89%) for classifying both intact and damaged building areas. As a result, the proposed method yielded satisfactory results in building damage detection. However, we still noted a few errors in several regions, with some typical error examples shown in Figure 11. For instance, even though some white areas in Figure 11a were located in the damaged buildings and belonged to the damaged roof areas, they were not detected. We suggest that the main reason for these errors is that these regions formed part of the building structure with a large area, and the color or geometric features were similar to the intact areas. Hence, these areas were identified as non-damaged ones. Some damaged facade areas were left undetected likely due to the insignificant damage type, and the selected features were used to discriminate them from the intact building areas. In terms of time consumption, detecting building damage in Scene II cost more time compared with in Scene I. The total number of points and different damage types may be the main reasons causing the difference in time consumption.

### 5.3. Comparative Analysis

#### 5.3.1. Comparison of Different Methods for Building Point Extraction

**Figure 12:**
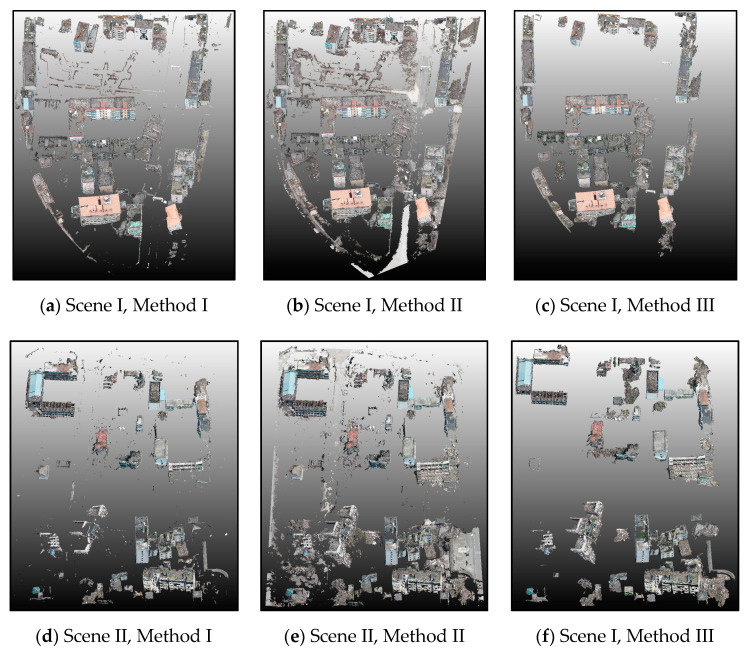
Performance comparison between different methods for building extraction.

To demonstrate the advantages of our method in building point extraction, we compared our method with the other three methods. The first method (Method I) was based on the RF algorithm applied to combined radiometric and geometric features, following Sanchez et al. [41]. The classification was performed based on radiometric features (e.g., HSV information) and geometric features (e.g., height, slope, linearity, planarity, scatter, and eigenentropy). The second method (Method II) used a commercial software Agisoft PhotoScan to extract the building points. To illustrate the advantages of adopting the PMF algorithm in landcover classification, the third method (Method III) separated ground from non-ground points based on the commonly used normalized digital surface model (nDSM), where processing was similar to the proposed method [42]. The model comparison results are presented in Figure 12. Referring to manually labeled reference data based on the visual interpretation results, Table 3 lists the comparison results using the different methods for building point extraction and taking both scenes (I and II) into account.

As shown in Figure 12 and Table 3, our proposed method performed the best in comparison with the three other methods. For Method I, the typical feature and sample selection determined the classification results to a large extent, making the process complicated and time-consuming. Moreover, the Method I final results included some noise, and it was unable to cover all of the collapsed building areas. Method II was characterized by high automation and time efficiency. Its final results covered all of the collapsed buildings to a certain extent. However, it included many extraction noises and falsely detected building points. Although Method III was distinguished by the high compactness of the final extracted building points, some debris around the collapsed buildings was misclassified as ground points, referring to the visual interpretation results. From final time consumption, the proposed method cost much more time compared to Method II and Method III. However, it was better to improve classification accuracy than costing much more time. In summary, the method proposed herein presents a balance of efficiency and accuracy. Even though the initial extraction of building points takes considerable time, the final results are highly accurate.

#### 5.3.2. Comparison of Different Methods for Building Damage Extraction

In this study, we proposed a combination of the supervoxel-based LDA and RF models to identify the damaged regions from building points. To prove the superiority of the proposed method and demonstrate its advantages, we used the following three methods for the comparative experiments:

(a) Supervoxel-based LDA and SVM classification (Method I): Based on the impact of different classifiers, we employed a support vector machine (SVM) to classify supervoxels using the generalized supervoxel features.

(b) Point-based RF classification (Method II): Point-based approaches commonly have a low recognition rate owing to the noise. To illustrate the influence of different basic units on damage classification, we used each discrete point as a classification object. We then integrated different features to compose the feature vectors. The discrete points were later classified using the RF classifier.

(c) Supervoxel-based BOVW and RF classification (Method III): Different feature representations of supervoxels were also a key issue, which directly influenced the classification accuracy. By employing the feature constructed by BOVW, instead of our LDA-based features for the supervoxels, we classified and labeled supervoxels using the RF classifier [34].

**Figure 13:**
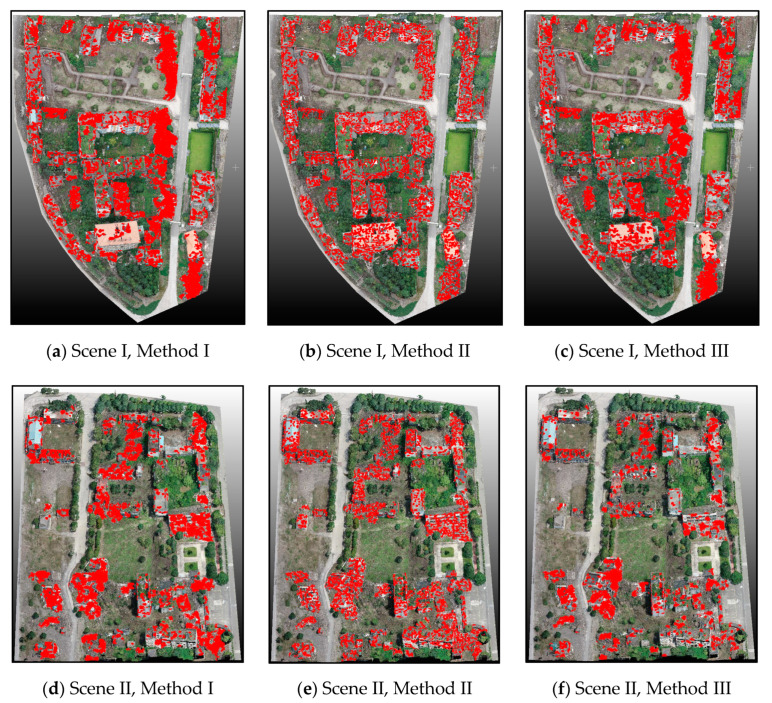
Performance comparison between different methods for building damage extraction.

The final damage detection results are shown in Figure 13, whereas the accuracy evaluation results are listed in Table 4. 

We performed both qualitative and quantitative evaluations based on the classification results for Scene I and Scene II. A comparison of the extraction results between the RF and SVM classifiers showed similar extraction performance, which can be reflected from the final approximate extraction accuracy (Table 4). As this paper focused on the application of the proposed method in 3D and not on distinguishing greater differences between the supervised methods, we conducted only one simple comparison experiment. The point-based and supervoxel-based strategies led to significantly different classification results (Figure 9b,e and Figure 12b,e). The application of point-based methods presented a significant ‘salt and pepper’ phenomenon, whereas the supervoxel-based method suppressed the ‘salt and pepper’ phenomenon, with remarkable improvements in OA of around 8% (particularly for classifying small objects). When we compared the extraction results for the LDA-based feature representation and the BOVW-based feature representation, pronounced differences existed in the extracted damaged regions. In contrast, the completeness of the LDA-based results was higher. As such, the LDA model was more sensitive to the specific classes and showed high accuracy for the extracted damaged regions, indicating its superiority. Compared with other methods, the proposed method cost less time, which suggested that LDA model promoted damage detection efficiency to some degree.

#### 5.3.3. Comparison of Different Features for Building Damage Extraction

**Figure 14:**
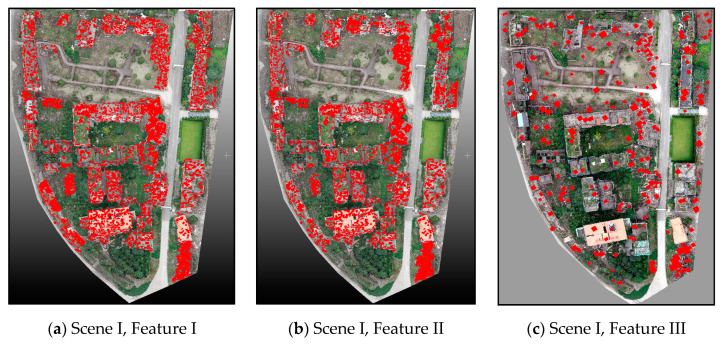

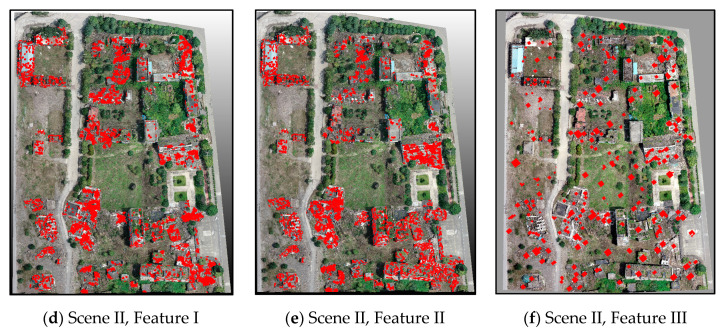
Performance comparison between different features for building damage extraction.

In this study, the proposed building damage detection method was based on the combined 2D and 3D features. To illustrate the difference in the usage of 2D and 3D damage features (the same ones as introduced in Section 3.3), we conducted a comparative experiment (Feature I and Feature II). In addition, to present the extraction difference in the CNN features, we also conducted damage extraction from the orthophotos of the test scenes (Feature III)—a commonly used method in the existing research. Herein, we conducted the experiment based on the well-known AlexNet model, which recognized for its high performance in object detection. The damage extraction results are presented in Figure 14, and the average evaluation accuracies are listed in Table 5. 

As can be seen in Figure 14, Features I and II caused higher levels of false extraction (some undamaged regions were misclassified as damaged regions) and missed extraction, which can also be reflected from the extraction accuracy (Table 5). Using 3D features for object detection tends to show better results than merely using 2D features. As for the commonly used CNN feature (Feature III), the final results presented the ‘salt-and-pepper’ effect, which significantly reduced the extraction accuracy. Furthermore, the CNN method was unable to preserve the abundant boundary information of damaged regions. Thus, using only 2D or 3D features was not sufficient for describing the actual damage situation, and the advantages of combining them is clearly demonstrated herein. As for the time consumption, Features I and II were more time-efficient than the proposed combined method due to fewer feature calculations and model training. In addition, Feature III took even more time to operate than the combined method due to its complicated model training and prediction. However, compared with time consumption, the improvement in the classification accuracy was more significant and important.

### 5.4. Parameter Sensitivity Analysis

#### 5.4.1. Majority Percent

**Figure 15:**
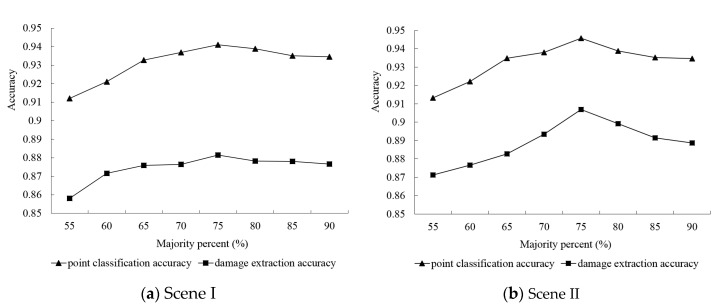
Parameter sensitivity analysis of majority percent for Scene I (**a**) and Scene II (**b**).

In the post-processing stage, the ground and vegetation data were first extracted at the pixel level. Then, the initial classification results were optimized based on the majority percent of the supervoxel. To analyze the impact of different majority percentages on post-processing results, the test majority percent was set from 55% to 90% at intervals of 5%. As shown in Figure 15, the variation in damage extraction accuracy was similar to that of image classification accuracy, indicating that the majority percent affected the final image classification and damage extraction. Nevertheless, its influence was limited (within 3%) likely due to the fact that the generated supervoxels (based on an improved VCCS algorithm) were individual, and most supervoxels could only contain one object class. As indicated in Figure 14, we set the optimal majority percent to 75% for ground and vegetation.

#### 5.4.2. Supervoxel Resolution

**Figure 16:**
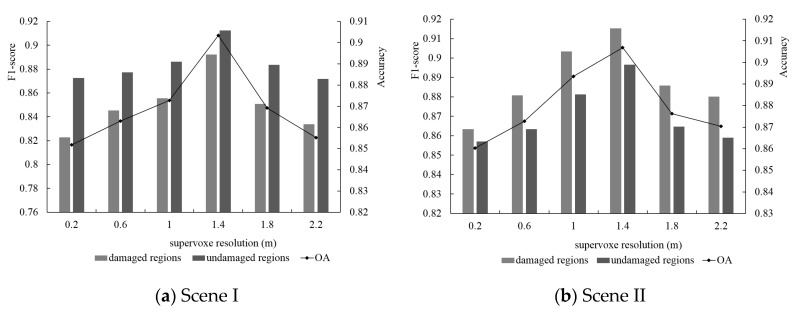
Parameter sensitivity analysis of supervoxel resolution for Scene I (**a**) and Scene II (**b**).

Supervoxel was used as a unitary element to classify the damaged and undamaged points. Thus, the number and shape of the supervoxel directly affected the final classification results. In this section, we explored the relationship between the supervoxel and classification results by changing the initial supervoxel resolution. F_1_-score and OA were used to evaluate the final performance of damage detection. As the histogram in Figure 16 illustrates, the F_1_-score over various objects changed as the supervoxel resolution increased. The line graph shows the effect of the supervoxel resolution on the classification results. Based on the overall accuracy, different supervoxel resolutions significantly influenced the final classification results, and the optimal value of the supervoxel resolution was determined to be 1.4 m. Concluded from the F_1_-score of the damaged and undamaged regions, the classification accuracy of the damaged regions was more influenced by the supervoxel resolution than that of the undamaged regions. Thus, both damage classification and the parameter sensitivity experiments were conducted based on the 1.4 m supervoxel resolution. 

#### 5.4.3. Latent Topic Number

**Figure 17:**
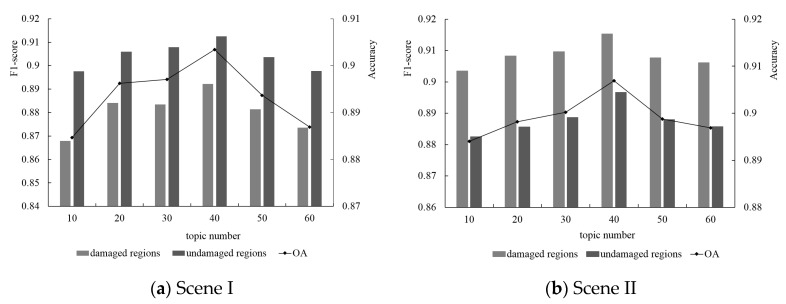
Parameter sensitivity analysis of latent topic number for Scene I (**a**) and Scene II (**b**).

For the LDA model, different numbers of latent topics may directly influence the classification performance. In this section, we used the point clouds from Scene I and Scene II to compare the damage classification results based on different numbers of topics. The histogram and the line graph in Figure 17 show the evaluation results based on the F_1_-score and overall accuracy, respectively. Different topic numbers influenced the final classification accuracy to a certain extent. However, compared with the influence of the supervoxel resolution, their influence was weaker, as concluded from the range of F_1_-score and overall accuracy. Moreover, the classification accuracy of the damaged regions was much more sensitive to the topic number than that of the damaged regions. When the number of ‘latent topics’ increased to 40, the highest accuracy was achieved.

#### 5.4.4. Visual Word Number

**Figure 18:**
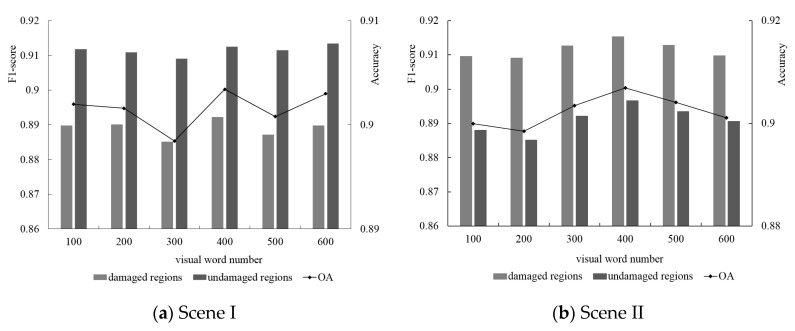
Parameter sensitivity analysis of visual word number for Scene I (**a**) and Scene II (**b**).

In this paper, we used the defined ‘visual word’ to generalize each supervoxel. ‘Visual word’ is the cluster center based on the feature vectors of the point cloud. The performance of the ‘visual word’ dictionary is determined directly by the number, n, of cluster centers. If the value of n is too small, two dissimilar points may be mapped to the same ‘visual word’, whereas if n is too large, two similar points might be mapped to different ‘visual words’. As shown in Figure 18, we analyzed the impact of different numbers of visual words on the final result based on the point clouds of Scene I and Scene II. Other parameters, such as the number of topics and the supervoxel resolution, were referred to as the optimal results. As shown in Figure 18, we set the optimal number of visual words to 400. 

#### 5.4.5. Number of Trees and Depth for RF Algorithm

**Figure 19:**
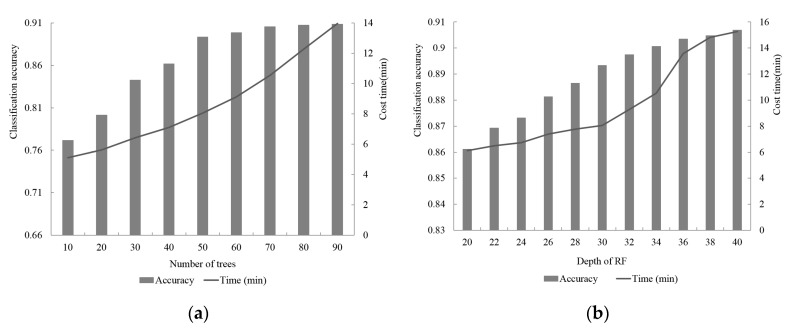
Performance comparison of different number of trees (**a**) and depth (**b**) for RF algorithm.

For the RF algorithm, the depth of RF and the number of trees directly affect the classification efficiency in damage detection. As shown in Figure 19 we conducted the parameter sensibility test for both of them. When the number of trees was less than 50, the damage classification accuracy rapidly increased. When the number of trees was over 50, the damage classification accuracy grew slowly and increased the cost of classification exponentially. Similarly, with the increase in depth for the RF algorithm, the classification accuracy and cost time presented a significant growth tendency. To maintain a good balance between accuracy and efficiency, the optimal number of trees and depth for the RF algorithm were set to 50 and 30, respectively.

### 5.5. Transferability Analysis of Other Areas

**Figure 20:**
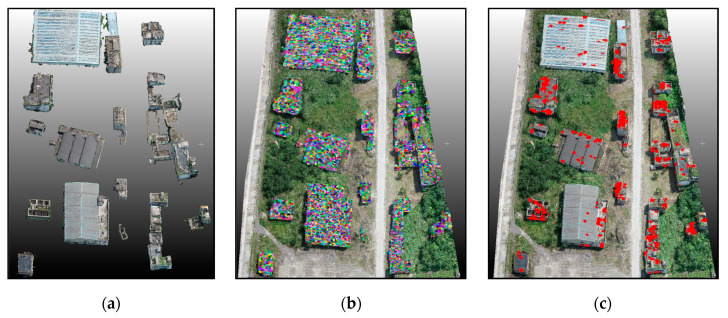
Results of the proposed framework for Scene III. (**a**) Extracted building points results; (**b**) boundary refined supervoxels of building points; (**c**) extracted damaged building regions.

To assess the transferability of the proposed framework, including building point extraction, building damage classification, obtained parameters, and trained classifier, we used Scene III with moderate damage for the building damage experiment. The final building results are shown in Figure 20. The evaluation indices, including precision, recall, and overall accuracy, are shown in Table 6. The proposed framework achieved overall accuracy of 0.92 for building point extraction and of 0.90 for building damage identification (Table 6). The overall extraction and classification accuracy for Scene III illustrated the good transferability of the proposed framework. 

## 6. Conclusions and Future Work

This study used the high-accuracy and semi-automated method to assess building structural damage by integrating 2D and 3D features. The entire process was conducted systematically, including model implementation of building point extraction, sample set construction, and model implementation of damage extraction. The proposed damage detection framework was additionally compared with other commonly used approaches. We verified its effectiveness through the transferability analysis in another scene. 

Considering its low cost and convenience, we used the UAV-based oblique photogrammetry technique to obtain dense building point clouds. As the final classification results in traditional methods tend to be easily affected by land-cover types, we proposed a systematic and detailed damage extraction framework. It included building point extraction and damage classification, which offered a balance between efficiency and accuracy. After confirming that the extraction results using traditional point-based methods suffered from the ‘salt-and-pepper’ effect, we proposed a supervoxel-based damage classification method. In contrast to the VCCS algorithm, we developed a boundary refined supervoxelization algorithm to improve the damage classification precision. Our proposed method also fully considered the 2D and 3D damage features of the building roof and facade using the LDA model for damage extraction. The proposed method improved the damage detection accuracy and the highest improvement ratio is over 8%. As determined by the quantitative analysis, the extraction accuracy of the building points reached approximately 94%, while the detection accuracy of building damage reached almost 90%. Moreover, both the precision and recall for damage detection reached 89%, illustrating both the reliability and accuracy of the proposed method. In terms of time consumption, the proposed LDA model promoted the damage detection efficiency compared with the classic model. In conclusion, the new building damage detection framework is based on the 3D analytical method and is convenient for a post-disaster emergency, meeting the need for accuracy and efficiency under emergency response.

In future studies, we plan on expanding the data source onto various post-disaster areas. With the use of additional damage samples, we can not only verify the further transferability of the proposed method but also integrate other types of building damage characteristics to help determine a specific damage level. Considering different manually selected damage features can significantly affect the final damage detection results, we must find those discriminative and representative high-level features to conduct building damage classification. With the development of 3D deep learning, increasing focus on 3D object recognition has motivated more research to conduct related studies. However, owing to the limited number of damage samples and complex building damage features, existing 3D recognition models are insufficient to cope with building damage detection. Thus, a more effective 3D detection method for building damage needs to be developed.

## Figures and Tables

**Table 1:** Accuracy evaluation results of building extraction.

	Scene 1		Scene 2	
	Pre.	Rec.	Pre.	Rec.
Building	0.9416	0.9227	0.9525	0.9232
Non-building	0.9408	0.9555	0.9406	0.9635
OA	0.9411		0.9457	
TIME (min)	10.11		10.25	

**Table 2:** Accuracy evaluation of proposed methods in different damage scene.

	Scene 1		Scene 2	
	Pre.	Rec.	Pre.	Rec.
Damage	0.8622	0.8664	0.9026	0.9284
Non-damage	0.8964	0.8931	0.9125	0.8813
OA	0.8814		0.9069	
TIME (min)	7.22		8.90	

**Table 3:** Performance comparison for building point extraction using different methods.

	Method I		Method II		Method III		Proposed Method	
	Pre.	Rec.	Pre.	Rec.	Pre.	Rec.	Pre.	Rec.
Building	0.8627	0.8044	0.8526	0.8391	0.9236	0.8905	0.9471	0.9229
Non-building	0.8348	0.8853	0.8713	0.8824	0.9148	0.9411	0.9407	0.9595
OA	0.8471		0.8631		0.9186		0.9434	
TIME (min)	16.85		6.26		9.30		10.18	

**Table 4:** Performance comparison for building damage detection using different methods.

	Method I		Method II		Method III		Proposed Method	
	Pre.	Rec.	Pre.	Rec.	Pre.	Rec.	Pre.	Rec.
Damage	0.8758	0.8801	0.8158	0.7998	0.8561	0.8441	0.8835	0.8987
Non-damage	0.8837	0.8795	0.8009	0.8168	0.8427	0.8574	0.9029	0.8882
OA	0.8798		0.8082		0.8498		0.8933	
TIME (min)	8.37		22.31		8.93		8.06	

**Table 5:** Performance comparison for building damage detection using different methods.

	Feature I		Feature II		Feature III		Combined Feature	
	Pre.	Rec.	Pre.	Rec.	Pre.	Rec.	Pre.	Rec.
Damage	0.7518	0.7264	0.7818	0.7861	0.8561	0.5829	0.5888	0.8987
Non-damage	0.7239	0.7494	0.7925	0.7883	0.6031	0.5972	0.9029	0.8882
OA	0.7377		0.7872		0.5931		0.8933	
TIME (min)	6.12		6.85		12.37		8.06	

**Table 6:** Accuracy assessment of building points and damage extraction for Scene III.

	Building Point Extraction		Building Damage Extraction	
	Building	Non-Building	Damage	Non-Damage
Pre.	0.9103	0.9216	0.8974	0.8996
Rec.	0.9121	0.9199	0.8860	0.9098
OA	0.9163		0.8986	
